# Cold-Induced Male Meiotic Restitution in *Arabidopsis thaliana* Is Not Mediated by GA-DELLA Signaling

**DOI:** 10.3389/fpls.2018.00091

**Published:** 2018-02-05

**Authors:** Bing Liu, Nico De Storme, Danny Geelen

**Affiliations:** ^1^Department of Plant Production, Faculty of Bioscience Engineering, University of Ghent, Ghent, Belgium; ^2^School of Integrative Plant Science, Cornell University, Ithaca, NY, United States

**Keywords:** GA signaling, cold stress, male meiotic restitution, meiotic cytokinesis, diploid pollen

## Abstract

Short periods of cold stress induce male meiotic restitution and diploid pollen formation in *Arabidopsis thaliana* by specifically interfering with male meiotic cytokinesis. Similar alterations in male meiotic cell division and gametophytic ploidy stability occur when gibberellic acid (GA) signaling is perturbed in developing anthers. In this study, we found that exogenous application of GA primarily induces second division restitution (SDR)-type pollen in Arabidopsis, similar to what cold does. Driven by the close similarity in cellular defects, we tested the hypothesis that cold-induced meiotic restitution is mediated by GA-DELLA signaling. Using a combination of chemical, genetic and cytological approaches, however, we found that both exogenously and endogenously altered GA signaling do not affect the cold sensitivity of male meiotic cytokinesis. Moreover, *in vivo* localization study using a GFP-tagged version of RGA protein revealed that cold does not affect the expression pattern and abundance of DELLA in Arabidopsis anthers at tetrad stage. Expression study found that transcript of *RGA* appears enhanced in cold-stressed young flower buds. Since our previous work demonstrated that loss of function of DELLA causes irregular male meiotic cytokinesis, we here conclude that cold-induced meiotic restitution is not mediated by DELLA-dependent GA signaling.

## Introduction

The production of viable haploid gametes is vital for the fertility and ploidy stability of flowering plants. Under certain conditions, plants may produce unreduced male gametes through incomplete meiotic cell division, a phenomenon termed ‘meiotic restitution’ or ‘meiotic non-reduction’ (Adams and Wendel, 2005;Mason and Pires, 2015). Meiotic restitution can be induced by omission of meiotic cell cycles, defective spindle organization, and/or irregular meiotic cytokinesis (De Storme and Geelen, 2013a; De Storme and Mason, 2014). Meiotic restitution can be classified into either FDR or SDR according to the genetic make-up of the yielded unreduced gametes (Kohler et al., 2010). In FDR-type meiotic restitution, homologous chromosomes fail to separate whereas sister chromatids successfully disjoin from each other. As a result, FDR-type unreduced gametes maintain parental heterozygosity in genomic regions where meiotic recombination occurs rarely. In contrast, in SDR-type meiotic restitution, homologous chromosomes segregate but sister chromatids are grouped into unreduced gametes, which typically leads to heterozygosity at recombination-free chromatin regions (De Storme and Geelen, 2013b). Meiotic restitution and associated formation of unreduced gametes can be induced either through specific genetic defects or by temperature stress. In *Arabidopsis thaliana*, for example, cold stress causes male meiotic restitution (primarily SDR-type) by specifically disrupting the organization of RMAs at the end of male meiosis, consequently resulting in incomplete meiotic cytokinesis and unreduced gamete formation (De Storme et al., 2012).

The phytohormone GA regulates multiple development processes during male reproduction in plants (Plackett et al., 2011; Kwon and Paek, 2016; Plackett and Wilson, 2016). In most cases, GA regulates plant development by suppressing the activity of DELLAs, a family of transcriptional repressors that operate as negative regulators of GA signaling (Sun, 2010). DELLA proteins negatively regulate the expression of GA-signaling downstream genes and consequently inhibit plant growth and development (Xu et al., 2014). At the molecular level, GA suppresses DELLA activity by promoting DELLA protein destabilization through 26S proteasome. GA-dependent DELLA degradation relies on activity of two F-box proteins SLEEPY1 (SLY1) and SNEEZY (SNE), and the phosphorylation status of DELLA itself (Ariizumi et al., 2011; Ariizumi and Steber, 2011; Qin et al., 2014). In Arabidopsis, there are five DELLA homologs; i.e., RGA, GAI, RGL1, RGL2, and RGL3.

During male reproductive development, programmed degradation of the tapetal cell layer during male gametogenesis and pollen maturation strongly relies on balanced GA signaling and alterations herein lead to defects in tapetal disintegration, eventually causing abortion of microspores or mature pollen grains (Cheng et al., 2004; Aya et al., 2009; Plackett et al., 2014). GA signaling also plays a role in one or more process of microsporogenesis; e.g., meiotic cytokinesis. Ectopic activation of GA signaling in Arabidopsis meiosis-stage anthers, either via exogenous GA treatment or in the DELLA *rga^-/-^ gai^-/-^* mutant background, was recently found to cause defects in male meiotic cell wall formation, leading to ectopic events of male meiotic restitution and associated formation of unreduced (2n) spores (Liu et al., 2017b). These findings suggest that balanced GA levels and associated GA signaling in the tapetal cell layer contributes to the progression of male meiotic cell division and is required for gametophytic ploidy stability (i.e., haploid spores). As the cellular mechanism of GA-induced male meiotic restitution highly mimics the meiotic alterations induced by cold (i.e., defects in RMA formation at telophase II and binuclear spore formation), we hypothesized that cold-induced male meiotic restitution in Arabidopsis could be mediated by GA-DELLA signaling. For this hypothesis to be correct, bioactive GA levels would need to increase in response to cold in the Arabidopsis anthers, in contrast to a general notion that low temperature stress causes a reduction in GA level and thereby promoting DELLA accumulation (Colebrook et al., 2014). In Arabidopsis seedlings, cold-induced accumulation of DELLAs is achieved by enhancing the catabolism of endogenous bioactive GA, through the rapid transcriptional activation of *DREB1b*/*C-repeat*/*DRE Binding Factor1* (*CBF1*), a positive regulator of GA-deactivating *GA2OX2* (Achard et al., 2008). Similarly, in rice, low temperature reduces the level of endogenous bioactive GA in developing anthers, where the expression of the GA biosynthesis gene *OsGA3OX1* is down-regulated upon cold stress, whereas the GA signaling repressor *SLR1*/*DELLA* and its upstream cold-responsive factor *CBF1* are up-regulated (Sakata et al., 2014).

Here, in this study, we test the hypothesis that cold-induced meiotic restitution in Arabidopsis male sporogenesis is mediated by alterations in anther GA signaling. In support of our hypothesis, we found that exogenous GA treatment of Arabidopsis primarily induces SDR-type unreduced gametes, in a similar rate and manner as when plants are exposed to cold. However, contrary to the assumption that GA may mediate the cold response of male meiosis, our data indicated that the cold sensitivity of male sporogenesis does not rely on the DELLA-dependent GA signaling. In addition, we found that cold stress does not reduce RGA abundance in the young developing anthers. Together these findings indicate that cold-induced male meiotic restitution in Arabidopsis is not mediated by GA-DELLA signaling.

## Materials and Methods

### Plant Materials and Growth Conditions

Arabidopsis (*Arabidopsis thaliana*) Columbia-0 and Landsberg *erecta* (L*er*) accessions were obtained from the Nottingham Arabidopsis Stock Centre. The GA-insensitive mutant *gai* was kindly shared by Patrick Achard. The DELLA double mutant *rga-24 gai-t6* was previously described (Achard et al., 2006) and were kindly provided by Patrick Achard and Nicholas Harberd. The fluorescent tagged lines (FTLs) in the *qrt1-2^-/-^* background (FTL1313 and FTL3332), used for genotyping unreduced gametes, were described earlier (Francis et al., 2007; Berchowitz and Copenhaver, 2008). FTL1313 marker is physically located at 11.2 cM on chromosome 1, and FTL3332 is positioned at 10.43 cM on chromosome 3^1^ (Supplementary Figure S1A) (Francis et al., 2007; Singh et al., 2017). The *pRGA::GFP-RGA* transgenic plants were obtained from Nicholas Harberd. Primers used for mutant genotyping are listed in Supplementary Table S2.

Seeds were germinated on K1 medium for 6–8 days and seedlings were transferred to soil and cultivated in growth chambers at 12 h day/12 h night, 20°C, and less than 70% humidity. To stimulate flowering transition, the photoperiod was changed to a 16-h-day/8-h-night regime. For GA_3_ and PAC treatment, flowering plants were sprayed by water (+0.02% Tween), 100 μM GA_3_ (+0.02% Tween) and 1 mM PAC (+0.02% Tween), respectively. The chemical concentrations used in this study were chosen based on previous study (Liu et al., 2017b).

### Measurement of Cold Sensitivity of Arabidopsis Sporogenesis

Young flowering Arabidopsis plants were treated with cold (4°C–5°C for 48 h) and the unicellular stage microspores were examined at 24–36 h after the cold treatment under microscope. The cold sensitivity of the *rga-24 gai-t6* mutant was analyzed in the *qrt1-2^-/-^* background. The microspores were released by squashing targeted stage buds on a microscope slide with a drop of orcein staining buffer. The assessment of unreduced microspores was done by comparing the size to the haploid microspores and/or by counting the number of nucleus. The cold sensitivity of sporogenesis was evaluated by quantifying the frequency of enlarged unicellular stage microspores among the haploid microspores in a same flower bud. For each plant individual, more than 500 meiotic products were counted for quantification; and if the total number could not reach 500, then all the meiotic products were counted. More than five biological replicates have been performed, and Student’s *t*-test was used for significance analysis at the 5% significance level (α = 0.05).

### GFP Intensity Quantification

To determine the effect of cold on abundance of GFP-tagged DELLA RGA proteins, Image J software was used for the quantification of GFP intensity. First, Image J was set up by selecting ‘SET MEASUREMENTS’ in ANALYZE menu with AREA, INTEGRATED DENSITY and MEAN GRAY VALUE being selected. Second, using any of the drawing/selection tools in Image J, the entire anther area of a tetrad stage anther picture taken from a *pRGA::GFP-RGA* plant without cold treatment, was chosen for fluorescence quantification by clicking ‘MEASURE.’ The value was recorded as integrated density (ID1). Thirdly, a region in the same anther where developing meiocytes were located was chosen as background and the fluorescence intensity was quantified and recorded as mean fluorescence of background readings (FoBR1). Afterward, integrated density in pictures of another four anthers from four non-treated *pRGA::GFP-RGA* plants, and five anthers from five cold-treated (4°C–5°C for 24 h) *pRGA::GFP-RGA* plants was quantified (values are recorded as ID2-10, respectively). The mean background readings were recorded as FoBR2-10, respectively. When all samples were finished, the corrected total cell fluorescence (CTCF) for each sample was calculated using formula: CTCFn = IDn- (area of selected cell × FoBRn). Student’s t-test was used for significance analysis with significance level (alpha) = 0.05.

### Cytology

Callosic cell wall staining and the analysis of the male meiotic products (tetrad-stage analysis by aniline blue and orcein staining) were performed as described previously (Liu et al., 2017b). Flowering Arabidopsis *qrt1-2^-/-^* plants segregating for the FTL1313 or FTL3332 pollen fluorescent marker were sprayed with 100 μM GA_3_, and the mature pollen grains were observed at 7–9 days following GA treatment. FDR/SDR genotyping of mature pollen grains in the FTL lines (FTL1313 and FTL3332) was performed by releasing mature pollen grains in a drop of pollen extraction buffer (0.5 M EDTA) on a microscope slide, and visualized under fluorescence microscope. Five plant individuals were analyzed for either the FTL1313 or FTL3332 reporter. Meiotic spreads were prepared as described previously (Liu et al., 2017b).

### Tubulin Immunolocalization

The alpha-tubulin immunolocalization was performed according to the method of De Storme et al. (2012) with minor modifications. Briefly, the time of the first and second digestions by enzyme mix were adjusted to 3 and 1.5 h, respectively.

### Expression Analysis

The young flowering Arabidopsis wild type Columbia-0 plants were treated with cold (4°C–5°C) for 0, 2, and 24 h, respectively, and the total RNA from young flower buds was isolated using the RNeasy Plant Mini Kit with additional on-column DNase I treatment (Qiagen). First-strand cDNA was synthesized using the GoScript^TM^ Reversion Transcription System Kit (Promega). Quantitative gene expression analysis was performed by qRT-PCR on a Stratagene MX3000 real-time PCR system using the GoTaq^®^ qPCR Master Mix (Promega). For each treatment group, young flower buds from ten plant individuals were collected, and mRNA was isolated and pooled for further use. Two more bulks of flower buds (also from ten plants for each bulk) were sampled, for a total of three biological replicates for each treatment. For each bulked mRNA sample, qPCR was performed twice, with three technical replicates for each gene being surveyed (six technical replicates in total). Data from the six assays were pooled and then analyzed. The Arabidopsis *ACTIN2* gene (AT3G18780) was used as the reference gene. The data for each tested gene have been normalized to the value of internal control *AtACTIN2*, and the value of 2 and 24 h cold-treated samples were compared with non-cold treated samples (0 h cold treatment). The relative expression fold-change is presented and was calculated using 2ˆdelta-delta Ct method. Significance analysis was performed on the relative expression fold-change using Wilcoxon rank test. Significance level (alpha) was 0.05. Primers used for specific amplification of targeted gene transcripts are listed in Supplementary Table S3.

### Microscopy

The microscopy performed in this study was according to the previous report (Liu et al., 2017b).

## Results

### GA Primarily Induces SDR-Type Male Meiotic Restitution

In Arabidopsis, recombination hotspots are distributed along entire chromatin except for centromeric regions (Salomé et al., 2011). To determine the type of GA-induced meiotic restitution in Arabidopsis, we analyzed segregation of the hemizygous centromere-linked FTL markers FTL1313 (dsRed – Chr. 1) and FTL3332 (YFP – Chr. 3) in the *quartet1-2^-/-^* (*qrt1-2^-/-^*) background. Because we have previously shown that GA treatment may induce around 5% meiotic restitution (Liu et al., 2017b), we here only quantified the number of meiotic restituted products and classified them into either FDR- or SDR-type (Supplementary Table S1). Under control conditions, plants hemizygous for either the FTL1313 or FTL3332 reporter construct produced tetrads in which two out the four pollen grains were GFP fluorescent reflecting regular segregation of these fluorescent markers (Supplementary Figure S1C). At 7–9 days post GA treatment, GA-sprayed plants hemizygous for FTL1313 produced abundant tetrads and, in addition, a small amount of triads with two regular-sized haploid pollen grains and one larger, unreduced pollen grain. When fluorescent expression in these pollen triads was exclusively confined to the unreduced pollen grain or to both haploid pollen grains, the triads most likely originate from SDR-type restitution (Supplementary Figures S1D,E). In contrast, when fluorescent expression in the triads occurred in both a haploid and an unreduced pollen grain, the triads most likely originated from FDR-type restitution (Supplementary Figure S1F). In addition, GA-treated plants also produced dyads in which homologous chromosomes harboring centromere-linked hemizygous FTL markers either segregate both to one single unreduced pollen (Supplementary Figure S1G) or each to one single unreduced pollen (Supplementary Figure S1H), indicating for either SDR- or FDR-type meiotic restitution, respectively. GA-induced dyads and triads in *qrt1-2^-/-^* plants hemizygous for the FTL1313 reporter were found to contain 75.4% homozygous and 24.6% hemizygous for the locus containing the FTL1313 reporter. For GA-treated *qrt1-2^-/-^* plants hemizygous for the FTL3332 marker, 92.1% appeared homozygous and 7.9% appeared heterozygous for the FTL3332 locus (Supplementary Figure S1B,I–L). The predominant homozygous status of unreduced microspores of both the FTL1313 and FTL3332 marker lines indicated that GA primarily induced SDR-type meiotic restitution. These results are similar to what has been reported for cold stress-induced male meiotic restitution (De Storme et al., 2012).

### Exogenous Modulation of Anther GA Content Does Not Influence the Cold Sensitivity of Arabidopsis Male Sporogenesis

**FIGURE 1:**
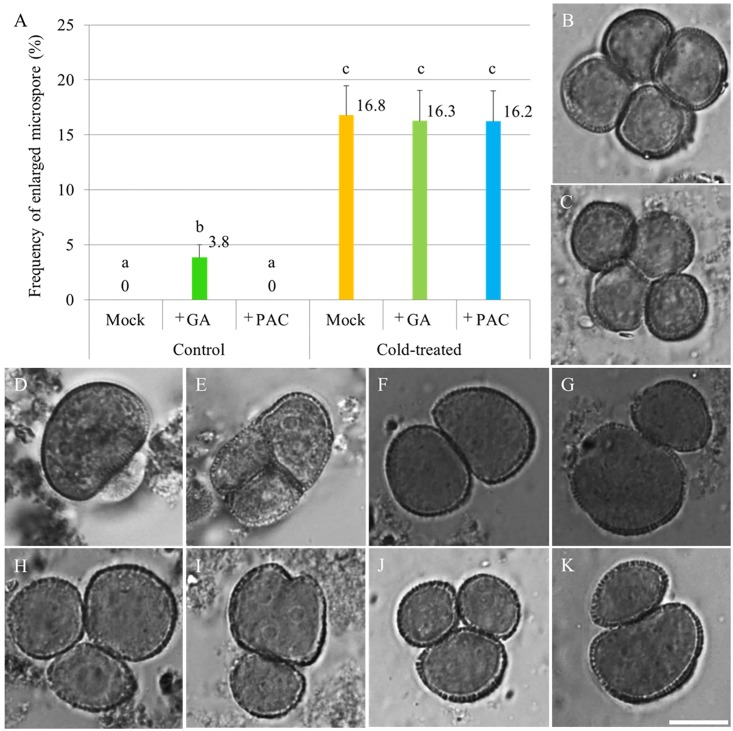
The effect of GA and PAC treatment on cold sensitivity of Arabidopsis male sporogenesis. **(A)** Histogram showing the frequency of enlarged unicellular microspores in *Arabidopsis thaliana* plants with combined chemicals and cold treatment. The numbers indicate mean values of the frequency of the enlarged microspores. For each group, 10 biological replicates were performed and more than 500 microspores have been counted. Student’s *t*-test was used for significance analysis. The *qrt1-2^-/-^* mutant was used for this quantification assay. **(B–E)** Haploid unicellular microspores in control plants **(B)**, plants treated with 1 mM PAC **(C)**, and enlarged unicellular microspores in plants treated with 100 μM GA_3_ **(D,E)** under normal temperature conditions. **(F–K)** Enlarged unicellular microspores in flowering plants exposed to a combined treatment of mock and cold **(F,G)**, GA and cold **(H,I)**, and PAC and cold **(J,K)**. Scale bar = 10 μm.

To test whether cold induces meiotic restitution by increasing endogenous GA level in anthers, flowering Arabidopsis plants were sprayed either with 100 μM GA_3_ or 1 mM of the GA biosynthesis inhibitor PAC, after which they immediately were transferred to cold conditions (4–5°C) for 48 h. 24–36 h post cold treatment, unicellular stage microspores were examined for indirect quantification of male meiotic restitution (**Figure 1**). Under normal temperature conditions, both mock and PAC-treated plants produced 100% uniformly sized haploid microspores (**Figure 1B**, mock-treated; C, PAC-treated). In contrast, GA_3_-sprayed *qrt1-2*^-/-^ plants produced around 3.8% enlarged microspores (**Figures 1A,D,E**). When an additional cold treatment was applied, mock, GA_3_ or PAC sprayed plants displayed similar frequency of enlarged unicellular microspores (**Figures 1A,F–K**). These data indicate that cold-induced meiotic restitution is neither enhanced nor reduced by exogenous GA and PAC application, suggesting that GA homeostasis is not critical for evoking a cold response in male meiosis.

### Genetic Alteration of GA Signaling Does Not Influence Sensitivity of Male Sporogenesis to Cold

**FIGURE 2:**
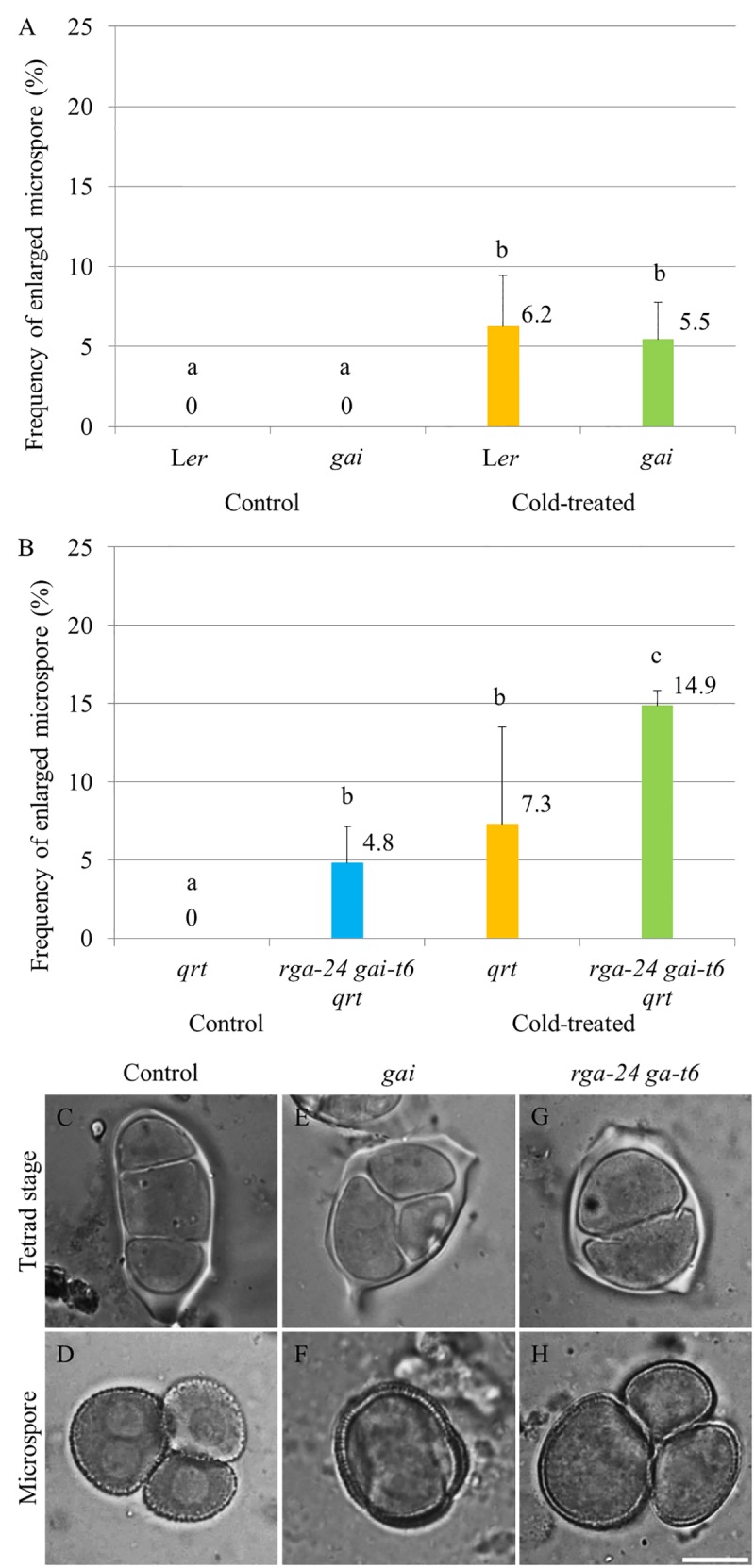
Cold sensitivity of male sporogenesis in Arabidopsis *gai* and *rga-24 gai-t6* mutant plants. **(A,B)** Histograms showing frequencies of cold-induced enlarged unicellular microspores in *gai* **(A)** and *rga-24 gai-t6***(B)** plants. The numbers indicate mean values of the frequency of the enlarged microspores. For each group, more than five biological replicates were performed and more than 500 microspores have been counted. Student’s *t*-test was used for significance. The *rga-24 gai-t6* mutant plants were in *qrt1-2^-/-^* mutant background. **(C–H)** Cold-induced male meiotic restitution and enlarged unicellular microspores in wild type L*er* **(C,D)**, the *gai* **(E,F)** and *rga-24 gai-t6 qrt1-2^-/-^***(G,H)** plants. Scale bar = 10 μm.

The impact of endogenous genetic alterations in GA signaling on the cold sensitivity of male sporogenesis was investigated by testing the cold response of both dominant and loss of function *della* mutant plants (**Figure 2**). The GA dominant insensitive *gai* mutant produces a non-degradable DELLA GAI protein and thus exhibits a constitutively repressed DELLA-dependent GA signaling (Peng et al., 1997). Under normal temperature conditions, the *gai* mutant produced normal tetrads and haploid microspores (Supplementary Figures S2C,D) as wild type plants (Supplementary Figures S2A,B). Upon exposure to cold (4–5°C for 48 h), *gai* plants exhibited male meiotic restitution with associated unreduced microspore formation at a similar level observed in wild type L*er* plants (**Figures 2A,E,F**), indicating that impaired DELLA-dependent GA signaling does not block the cold sensitivity of Arabidopsis male meiotic cell division. The double *rga-24 gai-t6* mutant plants exhibit constitutively activated GA signaling (Dill and Sun, 2001), and produced 4.8% unreduced male gametes under normal temperature conditions (**Figure 2B**). Cold was found to induce a significantly higher level of enlarged microspores compared to wild type plants (**Figures 2B–D**, wild type L*er*; G and H, *rga-24 gai-t6*), suggesting an additive effect of cold stress on meiotic restitution in the *della* null mutation background. These data demonstrate that the cold sensitivity and response of Arabidopsis male sporogenesis does not rely on RGA- and GAI-dependent GA signaling.

### Cold Induces Defective Male Meiotic Cytokinesis in the GA-Insensitive Mutant

We further examined male meiotic chromosome behavior and male meiotic cell wall formation in the GA-insensitive *gai* meiocytes. Cold stress did not disturb male meiotic chromosome segregation in both the *gai* mutant and wild type L*er* plants (Supplementary Figures S3A–C). Under normal conditions, *gai* displayed regular callosic cell wall formation indicating normal meiotic cytokinesis (Supplementary Figures S3D,G). However, at 24 h after cold treatment (4–5°C), meiotic-restituted dyads and triads with incomplete callosic cell walls were observed in both L*er* and the *gai* mutant (Supplementary Figure S3E,F,H,I) manifesting defective male meiotic cytokinesis.

**FIGURE 3:**
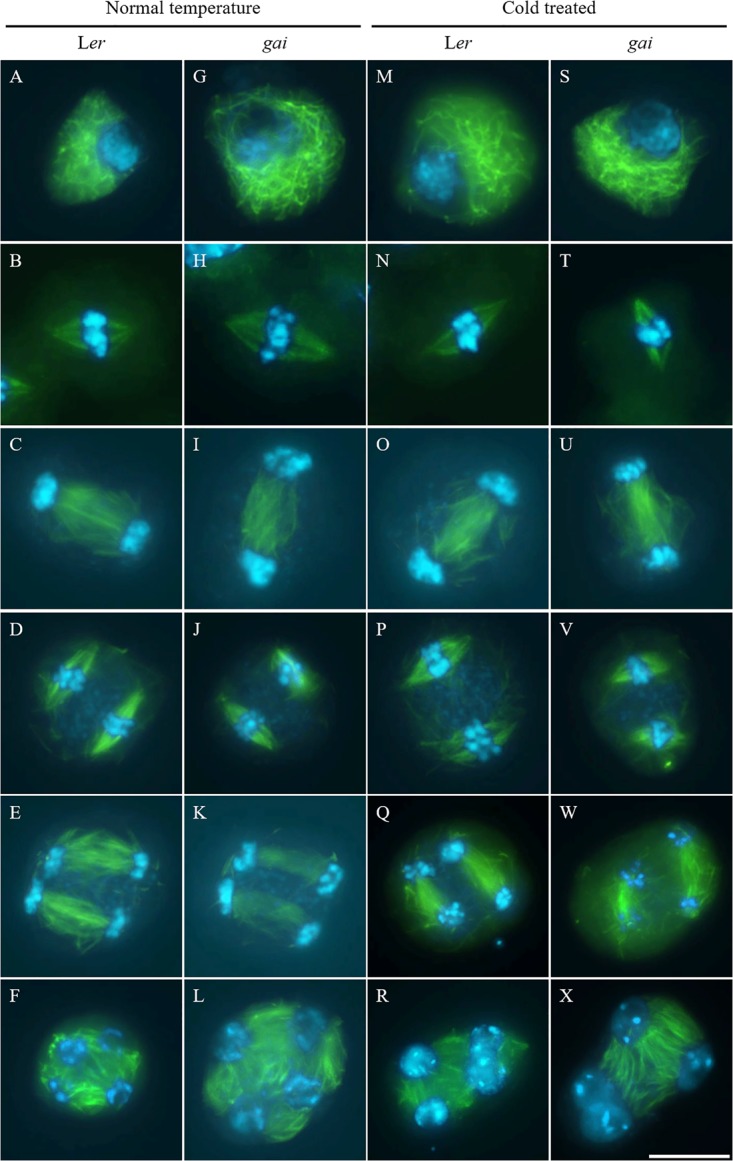
Cold interferes with radial microtubule array (RMA) formation in the *gai* mutant. **(A–L)** Meiotic microtubule structures at prophase **(A,G)**, metaphase I **(B,H)**, interkinesis **(C,I)**, metaphase II **(D,J)**, anaphase II **(E,K)** and telophase II stage **(F,L)** meiocytes in wild type L*er* **(A–F)** and the *gai* mutant **(G–L)** plants under control conditions. **(M–X)** Meiotic microtubule structures at prophase **(M,S)**, metaphase I **(N,T)**, interkinesis **(O,U)**, metaphase II **(P,V)**, anaphase II **(Q,W)** and telophase II stage **(R,X)** meiocytes in cold-stressed wild type L*er* **(M–R)** and the *gai* mutant **(S–X)** plants. Green: α-tubulin, cyan: DAPI. Scale bars = 10 μm.

Tubulin immunostaining was performed on the cold-shocked L*er* and *gai* mutant male meiocytes (**Figure 3**). During the stages from prophase I to anaphase II, both the control and cold-stressed meiocytes in either L*er* or *gai* plants displayed regular microtubule configurations (**Figures 3A–E,G–K,M–Q,S–W**). At prophase I, a network of microtubules surrounded the nucleus. Metaphase I showed formation of a single spindle, while metaphase II showed two perpendicular spindle formations. In contrast, the microtubule network; i.e., RMA, was clearly affected by cold at telophase II. Some of the nuclei in cold-stressed L*er* and *gai* tetrad were adjacent to each other and were not separated by RMA (**Figures 3R,X** and Supplementary Figure S4). These data demonstrate that the cold-sensitivity of meiotic cytokinesis and RMA in male meiocytes does not rely on DELLA-dependent GA signaling.

### Cold Does Not Reduce RGA Abundance in Tetrad-Stage Arabidopsis Anthers

**FIGURE 4:**
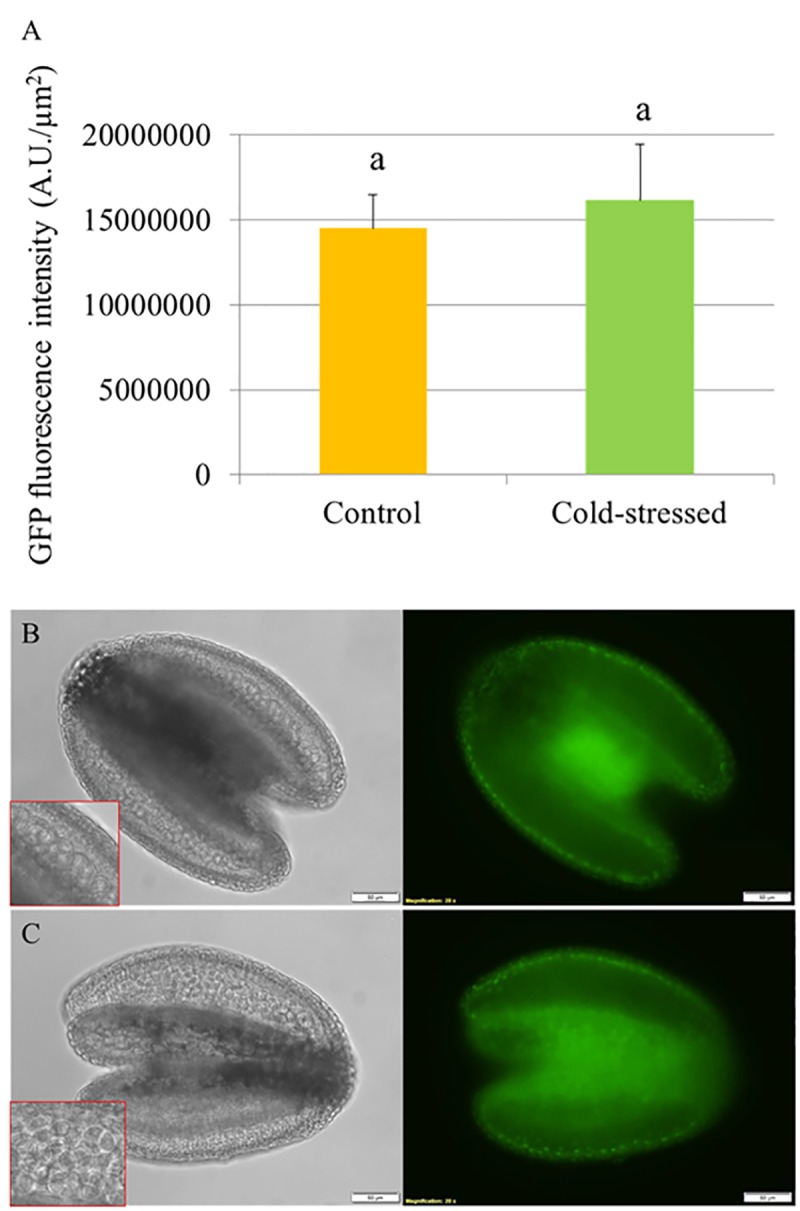
Cold stress does not reduce the abundance of GFP-RGA in tetrad stage Arabidopsis anthers. **(A)** Histogram showing the GFP fluorescence intensity in tetrad stage anthers under cold stress. Five biological replicates were performed and Student’s *t*-test was used for significance analysis with significance level (alpha) = 0.05. **(B,C)** Expression of *pRGA::GFP-RGA* in tetrad stage anthers under normal temperature conditions **(B)** and at 24 h under cold stress **(C)**.

To determine the effect of cold stress on abundance of DELLA proteins in developing Arabidopsis anthers, we used an Arabidopsis transgenic line harboring a recombinant *pRGA::GFP-RGA* reporter construct and monitored the *in vivo* GFP fluorescence signals in the developing anthers at 24 h under cold shock (4–5°C for 24 h). The abundance of RGA proteins was determined by quantifying the GFP intensity in the anthers (**Figure 4A**). If cold stress induces defective meiotic cytokinesis by promoting DELLA degradation, the fluorescence signals of GFP-RGA in tetrad stage anthers should be reduced. We observed that the *pRGA::GFP-RGA* was predominantly expressed in the tapetal cell layer and was not affected by 24 h cold stress (**Figures 4B,C**), suggesting that cold induces meiotic restitution in Arabidopsis anthers not by interfering with DELLA protein stability.

### The Expression of GA Metabolic and Signaling Genes in Young Arabidopsis Flower Buds Are Influenced by Cold Stress

**FIGURE 5:**
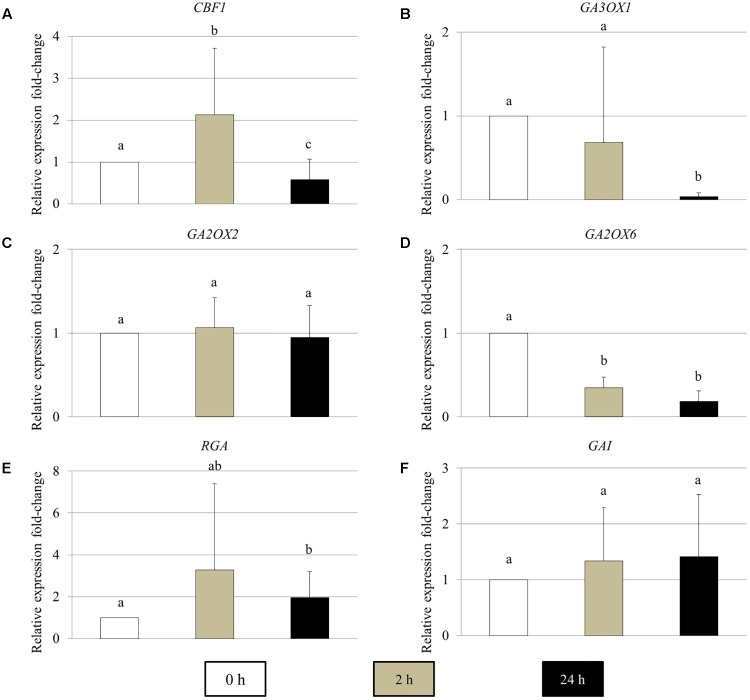
The relative expression fold-change of GA metabolic and signaling genes in Arabidopsis young flower buds under cold stress. **(A–F)** Histograms showing the relative expression fold-change of *CBF1* **(A)**, *GA3OX1* **(B)**, *GA2OX2* **(C)**, *GA2OX6* **(D)**, *RGA* **(E)**, and *GAI* **(F)** in early stage Arabidopsis flower buds under cold stress. *X* axis represents time period under cold treatment; *Y* axis represents relative expression ratio of the genes. Error bars represent standard deviation with significance level (alpha) = 0.05.

To determine the effect of cold stress on the expression of GA metabolic and signaling genes in young Arabidopsis flower buds, real-time quantitative PCR of the genes encoding for CBF1, GA biosynthesis GA3OX1, GA 2-oxidases GA2OX2 and GA2OX6, DELLA RGA and GAI proteins was performed (**Figure 5**). In 2 h cold-stressed young flower buds, the *CBF1* and *GA2OX6* transcript levels showed a significant increase and reduction compared to control plants without cold stress, respectively (**Figures 5A,D**), while for *GA3OX1* and *RGA*, no significant alterations were detected (**Figures 5B,E**). The transcripts of *GA2OX2* and *GAI* appeared stable throughout the cold treatment (**Figures 5C,F**). At 24 h under cold stress, the relative expression of *CBF1* and *GA3OX1* declined significantly (**Figures 4A,B**), contrary to *RGA* that showed an elevated expression level (**Figure 5E**). These data suggest that cold stress has an impact on the transcription of both GA synthesis and catabolic genes, and it modulates GA levels in Arabidopsis anthers in a complex manner.

## Discussion

Since cold stress and exogenous GA application induce male meiotic restitution using highly similar mechanism in Arabidopsis (De Storme et al., 2012; Liu et al., 2017b), we hypothesized that cold-induced defective male meiotic cytokinesis may be mediated by GA-DELLA signaling. We found that both of these treatments induce SDR-type unreduced pollen grains. This predominant restitution pathway is reminiscent to omission of second division or the elimination of the second meiotic division occurring in mutants defective in *CYCA1;2*/*TAM* and *OSD1* gene function. In these mutants, the second division is not executed because meiocytes exit the division cycle and immediately pursue with cell wall formation (d’Erfurth et al., 2010; Cromer et al., 2012). Cold and GA-induced restitution are clearly not the result of omission of second division. Instead they cause defects in the organization of the RMAs formed at the end of the second division cycle, with subsequent defects in cell plate formation. Microtubules between the haploid nuclei in tetrad stage meiocytes are poorly developed or maintained, allowing the nuclei to occasionally migrate into close proximity. Adjacent nuclei may then operate as a single unit during cell wall positioning and cytokinesis, establishing triad and dyad formation. Hence it is not likely that cold- and GA-induced cytokinesis defect is mediated by CYCA1;2/TAM and/or OSD1.

Contrary to our hypothesis, both endogenous and exogenous alterations in GA signaling did not influence the cold sensitivity of microsporogenesis, and cold stress did not reduce RGA-GFP abundance in tetrad stage anthers. In addition, the transcript levels of GA biosynthesis gene *GA3OX1* was downregulated and *RGA* was increased upon cold stress in young flower buds. These findings suggest that the cold sensitivity of male meiosis does not rely on a DELLA-dependent GA signaling. Since male meiotic cytokinesis is interfered by a reduced level of DELLAs, and not by an accumulation, we conclude that cold-induced male meiotic restitution in Arabidopsis is not mediated by GA-DELLA signaling.

Our gene expression analysis shows similarity with the study in rice, where low temperature has been shown to disrupt pollen development by lowering the bioactive GA level and increasing the abundance of DELLA protein in developing anthers, and displays a rapid increase of the *CBF1* transcript (Sakata et al., 2014). At the same time, we observed a negatively regulated expression of GA catabolic factor *GA2OX6* in addition to a downregulated expression of *GA3OX1*, exhibiting a similar situation in rice, in which cold stress reduces the expression of both GA catabolic gene *GA2OX1* and biosynthesis genes *OsGA20OX3* and *OsGA3OX1* (Sakata et al., 2014). These findings hint that reduced temperature modulates GA levels in male reproductive tissues by a complicated mechanism. Whether bioactive GA is reduced or upregulated would depend on a predominant impact of cold stress on GA biosynthesis, or catabolism, and may also depend on which cold-responsive GA metabolism gene plays a major role in the tetrad stage anthers. On the other hand, in Arabidopsis CBF1 and 3 inhibit vegetative development upon cold stress by enhancing the expression of *GA 2-oxidases* and increasing DELLA stability, and negatively regulating GA biosynthesis (Zhou et al., 2017). In both vegetative and reproductive tissues of plants, the expression of *CBF1* is rapidly increased upon cold stress and subsequently declines (Novillo et al., 2004, 2007; Achard et al., 2008; Sakata et al., 2014; Karimi et al., 2015). Our study here revealed a similar expression pattern of *CBF1* as these previous studies. Considering these findings, it is likely that the cold-responsive pattern of CBFs-GA-DELLA module might be conserved in different tissue types of multiple plants species.

Notably, although we observed that cold stress has an additive effect on 2n gamete formation in the *rga-24 gait6* plant background, we did not detect significant difference in plants with combined GA and cold treatment (**Figures 1**, **2**). This may because in the wild type plants, cold offset the effect of GA treatment by lowering GA levels in the anthers somehow, resulting in a non-significant destabilization of DELLAs. At the same time, although the expression of *RGA* increases in 24 h cold-stressed flower buds, we did not observe an elevation of GFP-RGA abundance at this time point under the cold condition (**Figures 4**, **5**). The construct used here may not reflect the endogenous RGA level or alternatively, cold stress differently induces responses of GA signaling in rice and Arabidopsis. Moreover, this fact may also be explained by the observation that although the cold stress reduced the expression level of GA biosynthesis gene *GA3OX1*, it also suppressed the activity of GA catabolic gene *GA2OX6*, which may consequently lead to a minor changed bioactive GA level that cannot significantly alter GFP-RGA abundance in the anthers. To determine a precise cold-responding behavior of bioactive GA levels, further studies should monitor GA gradients in the developing male reproductive tissues *in vivo* (Rizza et al., 2017). An additional difference with the study in rice anther is that whereas GA-insensitive rice mutants were hypersensitive to low temperature, exhibiting severe defects in pollen development and male fertility (Sakata et al., 2014), the Arabidopsis GA-insensitive *gai* mutant was not different from the control wild type and showed a similar cold-induced meiotic restitution. We conclude from these results that the meiotic restitution studied here is distinct from the cold-induced decrease in the number of sporogenous cells and hypertrophy of tapetal cells in rice, and GA signaling preferentially plays a role in later gametogenesis under cold stress.

GA has been shown to regulate cortical microtubule organization in epidermal cells of pea internodes and renders cortical microtubules more susceptible to cold (Akashi and Shibaoka, 1987). A possible mechanism involves the binding of DELLA protein to prefoldin complex, a chaperone required for tubulin folding, and its localization to the cytoplasm (Locascio et al., 2013). Under conditions that reduce GA levels, the prefoldin complex is targeted to the nucleus compromising tubulin heterodimer availability and affecting microtubule dynamics. Rice plant cells have been shown to respond to severe cold shock by modifying the cortical microtubular network and accumulation of microtubules at the nuclear envelope (Chen et al., 2011). The growth arrest in rice caused by the 4°C cold shock can be partially rescued by overexpressing OsRAN2 or TaRAN1, members of the small GTPase protein family that plays an important role in nucleo-cytoplasmic transportation of proteins and RNA, mitotic spindle assembly, and nuclear envelope assembly (Chen et al., 2011). The expression of RAN GTPases is subject to environmental stimuli and responds to different levels of exogenously applied plant hormones including GA (Chen et al., 2011; Tian et al., 2015). Hence, RAN protein function may be involved in cold-induced meiotic restitution and future studies should address the possibility that cold affects RAN-mediated re-localization of tubulin heterodimers. The hormone cytokinin also conveys cold responses in plants (Kazan, 2015; Eremina et al., 2016). We previously investigated and reported that the cytokinin signaling module AHK2/3-AHP2/3/5, which plays a role in the cold sensitivity and response of Arabidopsis vegetative development (Jeon et al., 2010; Jeon and Kim, 2013; Liu et al., 2017a), is not involved in cold response of meiotic cytokinesis. Whether cold-induced meiotic restitution is mediated by hormone controlled processes is currently not demonstrated and future studies may need to consider a more direct impact of cold on proteins involved in male meiotic cytokinesis.

## Author Contributions

BL performed the experiment and wrote the draft manuscript. BL and NDS conceived and designed the project. BL and NDS worked on manuscript edition. DG supervised the project, contributed to the experimental design and to the interpretation of results and edited the manuscript.

## Conflict of Interest Statement

The authors declare that the research was conducted in the absence of any commercial or financial relationships that could be construed as a potential conflict of interest. The reviewer NG and handling Editor declared their shared affiliation.

## Footnotes

1 https://www.arabidopsis.org/

## Abbreviations

FDR: first division restitution
GA: gibberellic acid
PAC: paclobutrazol
RMAs: radial microtubule arrays
SDR: second division restitution.
