# Supplementary material for: Cold-Induced Male Meiotic Restitution in Arabidopsis thaliana Is Not Mediated by GA-DELLA Signaling
Source: Front Plant Sci. 2018 Feb 5;9:91. doi: 10.3389/fpls.2018.00091 (PMC5807348; doi:10.3389/fpls.2018.00091)
Supplement: Supplementary file 1 [file Presentation_1.PDF]

Cold-Induced Male Meiotic Restitution in *Arabidopsis thaliana* Is Not Mediated by GA-DELLA signaling

Bing Liu[a, b], Nico De Storme[a], and Danny Geelen[a]*

[a]Department of Plant Production, Faculty of Bioscience Engineering, University of Ghent, 9000 Ghent, Belgium; [b]School of Integrative Plant Science, Cornell University, Ithaca, 14853 NY, United States

* correspondance: danny.geelen@ugent.be

<u>Supplemental figures</u>

Supplemental figure S1.

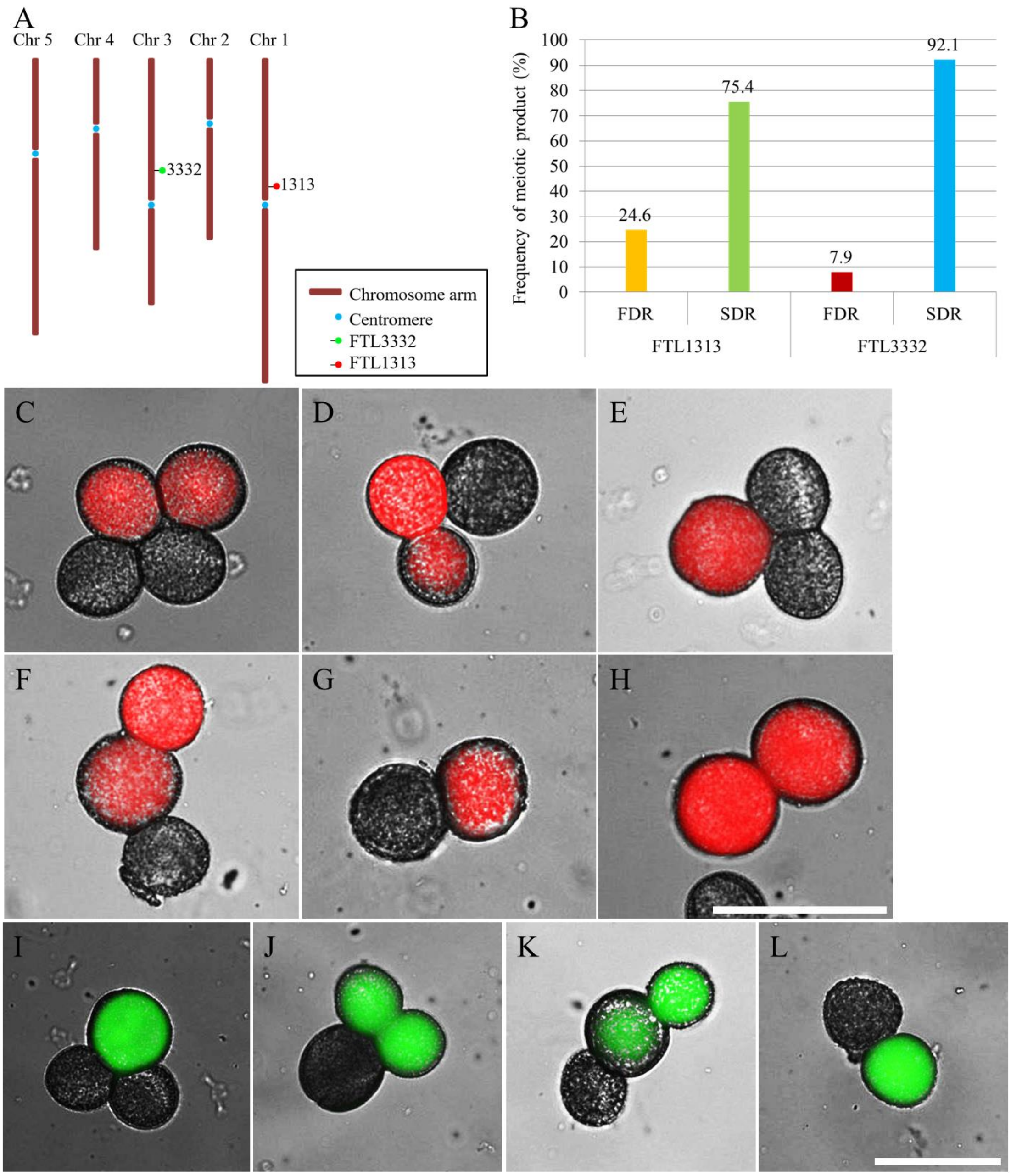

Supplemental figure S1. GA primarily induces SDR-type 2n male gamete in Arabidopsis. A, Genomic positions of FTL markers used in this study. B, Histogram showing the frequency of GA-induced meiotic restituted outcomes. Mean frequency numbers are indicated above the plotted bars. C-L, Characterization of GA-induced meiotic restituted pollen grains using *qrt1-2*$^{-/-}$ FTL markers (FTL1313, located as 11.2 cM on chromosome 1 expressing red fluorescent protein; FTL3332, located at 10.43 cM on chromosome 3 expressing yellow fluorescent protein). C, pollen tetrads reflecting regular 2:2 segregation of fluorescent markers. D-F, Meiotic restituted triads representing SDR-type (D and E) and FDR-type (F) pollen grains. G and H, Meiotic restituted dyads representing SDR-type (G) and FDR-type (H) pollen grains. I-K, Meiotic restituted triads representing SDR-type (I and J) and FDR-type (K) pollen grains in the FTL3332 line. L, Meiotic restituted dyads representing SDR-type (L) pollen grains in the FTL3332 line. The numbers of SDR and FDR restitution are presented in

Supplemental Table. S1. Scale bars = 50 µm.

Supplemental figure S2.

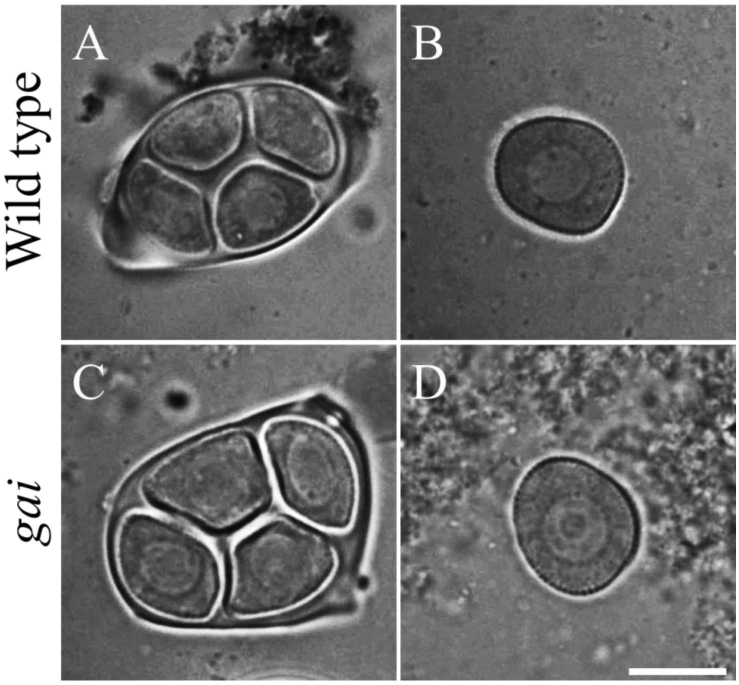

Supplemental figure S2. Sporogenesis of GA-insensitive *gai* mutant under the normal temperature conditions. A-D, Tetrads and haploid unicellular stage microspores in wild type L*er* (A and B) and *gai* (C and D) mutant plants. Scale bar = 10 µm.

Supplemental figure S3.

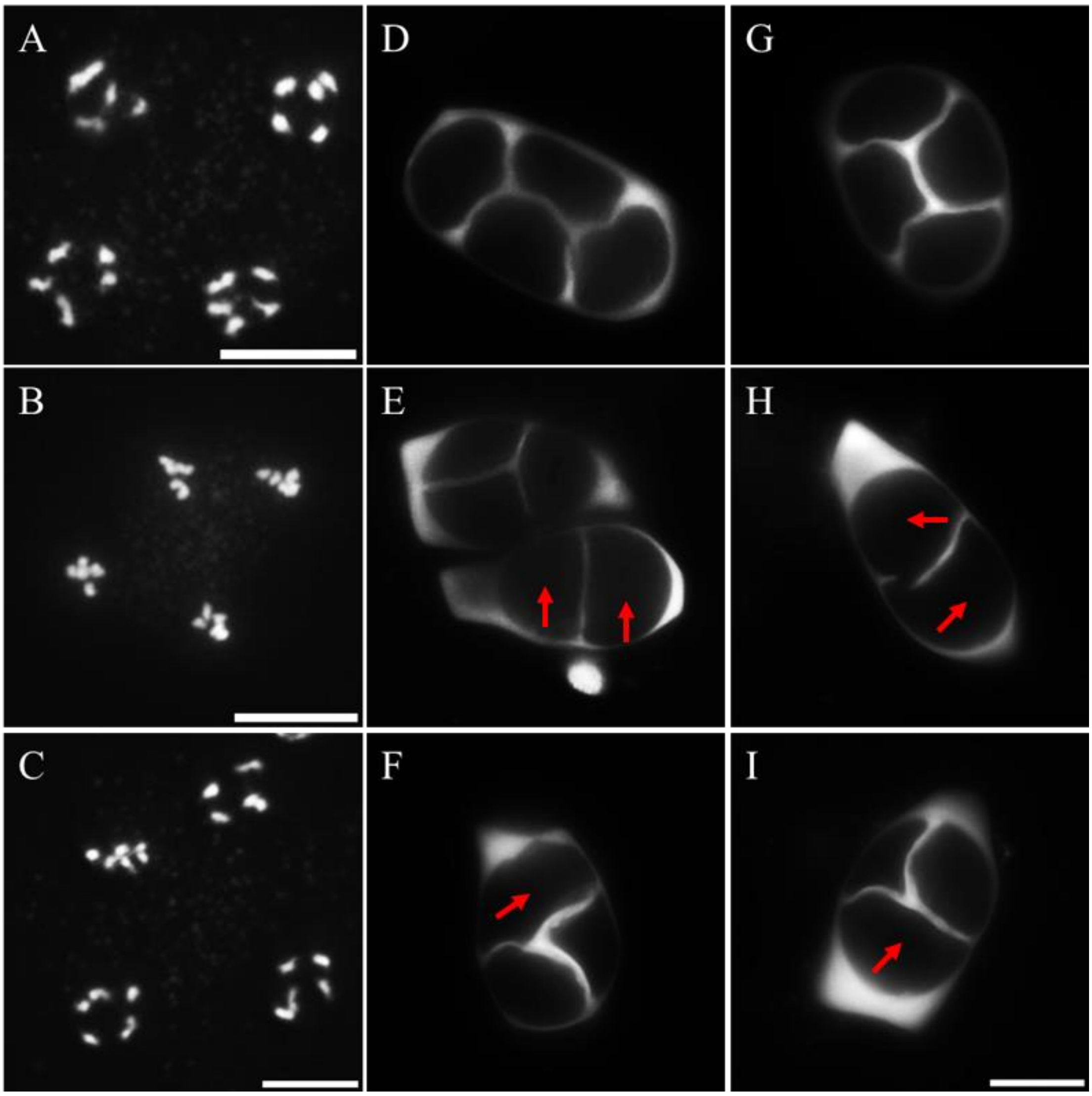

Supplemental figure S3. Cold interferes with meiotic cytokinesis in the GA-insensitive *gai* mutant plants. A-C, DAPI-stained male meiotic chromosome spreads of telophase II meiocytes in wild type L*er* plants under normal conditions (A), cold-stressed telophase II meiocytes in L*er* (B) and the *gai* mutant (C) plants. D and G, Aniline blue-stained callosic cell walls in tetrad stage male meiocytes of L*er* (D) and the *gai* mutant (G) plants under normal temperature conditions. E and F, H and I, Aniline blue-stained callosic cell walls in cold-stressed tetrad stage male meiocytes of L*er* (E and F) and the *gai* mutant (H and I) plants. Red arrows indicate incomplete meiotic cell walls. Scale bars = 10 μm.

Supplemental figure S4.

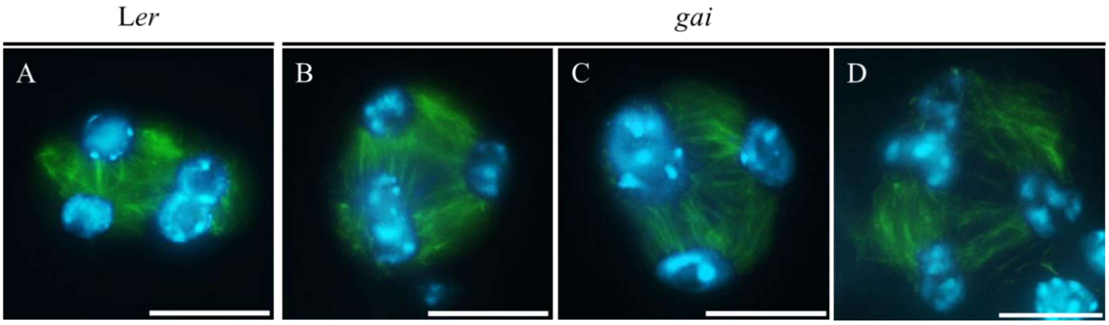

Supplemental figure S4. Cold affects the formation of the RMA configuration in the *gai* mutant. A-D, Triads in cold-treated wild type L*er* (A) and GA-insensitive *gai* mutant plants (B-D). Green: α-tubulin, cyan: DAPI. Scale bars = 10 µm.

Supplemental tables

Supplemental table S1.

Heterozygous FTL markers			Dyad		Triad		
Name	Label	Chr.	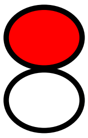	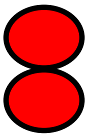	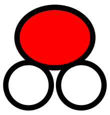	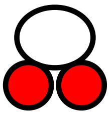	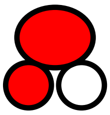
FTL1313	dsRed	1	18 (31.6%)	5 (8.8%)	13 (22.8%)	12 (21.0%)	9 (15.8%)
FTL3332	YFP	3	8 (21.1%)	0 (0%)	13 (34.2%)	14 (36.8%)	3 (7.9%)

Supplemental table S1. FTL-based genotypic characterization of GA-induced unreduced pollen. The numbers in columns of dyads and triads indicate the frequency of corresponding figures of meiotic-restituted pollen tetrads observed.

Supplemental table S2.

Target	genotyping primer sequence	
	forward	reverse
rga-24	TCGCTTAGTAGTTAGTACTC	CATAGACCATAGTATTCGTGA
gai-t6	TAGAAGTGGTAGTGG	GTGAACAGTCTCAGTAGC
gai	GCCCTCGTGCCCTTTTTATAC	CGACCGAAGCCAAACTAAATC

Supplemental table S2. Primers for mutant genotyping.

Supplemental table S3.

Transcript	Primer sequence (5’- 3’)	AT (℃)
ACTIN2-F	GGCTCCTCTTAACCCAAAGGC	51
ACTIN2-R	CACACCATCACCAGAATCCAGC	
GA3OX1-F	ATTTAGCTGGAGAGCAGCTTG	
GA3OX1-R	TTAAGTCTGCTCGGTCGGA	
GA2OX2-F	GGACCAAACGGTGACGTT	
GA2OX2-R	TATCCCTAGTTCTTCGGCAACC	
GA2OX6-F	GGGACAGAAGTCTAGCGAAGTG	
GA2OX6-R	CTAAACGGAGAGAGTATGTAGCTTTTT	
RGA-F	GAGTGTGCCAACCCAACAT	
RGA-R	AATCGAACCATAACGGACCG	
GAI-F	TGGCTAACACTTTAGCTGATCTTG	
GAI-R	CACCGCCGTTGAACAGAG	
CBF1-F	GCACCTTCGCTCTGTTCC	
CBF1-R	GCTCCGATTACGAGCCTCA	

Supplemental table S3. Primers for real-time Q-PCR.
